# Case of a Boy Whose Head Was Pressed between Certain Parts of an Engine Employed for Draining a Coal Mine

**Published:** 1792

**Authors:** Henry Yates Carter

**Affiliations:** Surgeon at Kettley, near Wellington, in Shropshire.


II. *Case of a Boy whose Head was pressed between certain Parts of an Engine employed for draining a Coal Mine. By the Same.*

ON the 21st of August, 1787, Thomas Scarrot, a boy about ten years of age, being at play, and looking into the cylinder at Hadley engine, (which is employed for draining a coal work) was caught between that part of it and what is called the horse head, and received a wound, which extended from the upper per part of the left temporal mufcle obliquely through the parotid gland and maffeter mufcle, dividing into two branches, one leading through the fphincter oris, the other paffing through the buccinator mufcle, and ending under the lower jaw.

The mufcular integuments were violently lacerated, the head having been preffed between a fpace of about three inches; and it feemed to be owing to this preffure that the hæmorrhage which might have been expected to take place was in a great meafure prevented.

The fore part of the os temporis was not only fractured, but completely feparated, as was the contiguous os malæ; the fracture continuing its courfe between the pterygoid and ftyloid proceffes, fo as to divide the upper jaw in thofe parts and the lower jaw a little before the coronal procefs. Thefe parts were divided in fuch a manner, that, when I firft faw the patient, I found the os malæ and the fore part of the upper jaw thrown out of their places, fo as to expofe a wound of at leaft four inches wide; the machine that inflicted it having alfo penetrated fo deep as to fracture that part of the feptum nafi which is formed by the proceffes of the ethmoidal and fphenoidal bones.

Befides

Befides thefe wounds, there was another upon the os bregmatis, which formed an angle that extended each way towards the os frontis, and had detached the fcalp the whole diftance fo completely, that when he was conveyed home the fcalp lay over his face.

Under thefe circumftances, which were the more alarming, as from the injury done to the parts neceffary to maftication and deglutition there was every reafon to fear that his fupport would be a work of much difficulty, there feemed to be but one confideration for the prefent with refpect to the wounds; and that was to endeavour, if poffible, to reduce the fhattered bones into their places, and there retain them as well as the nature of the cafe would admit, leaving the reft to nature, as any farther affiftance for the prefent could be but of very trifling effect.

The parts were accordingly firft well freed from a quantity of grumous blood that covered the whole head, by wafhing them with warm vinegar and a fmall proportion of camphorated fpirit of wine; after which the lacerated parts were replaced in a much better fituation than was at firft thought to be poffible. The lower jaw, which had been thrown out of its articulation on the right ſide, was reduced, and, after dreſſing the wounds with large pledgits of lint dipped in vinegar and *balſam. traumatic.* a maſk-like bandage was applied over the whole, and ſo ſecured behind as not to be in danger of removal from the expected reſtleſſneſs of the patient, as I determined to leave the dreſſings as long as poſſible before they ſhould be renewed.

In order to prevent the bandage, &c., from becoming uneaſy, and the parts from being excoriated, they were kept conſtantly wet with a mixture, conſiſting of a ſolution of ſal ammoniac in vegetable acid with a ſmall proportion of extract of lead, the good effects of which I had before experienced in lacerated wounds, where it had ſeemed to promote the union of the parts, and to moderate, in ſome meaſure, the diſcharge.

After the wounds were dreſſed, the patient attempted to drink a ſmall quantity of wine and water, but a conſiderable part of it returned through his noſe. In the evening, however, an opiate was got down without much difficulty, and he reſted tolerably well.

His drink was directed to be weak wine and water acidulated with lemon juice; his diet, thin thin panada, water gruel, ſago, or boiled rice: and, in conſequence of his inability to take much at a time, a perſon was employed to be frequently adminiſtering nouriſhment, which was conveyed into the pharynx by means either of a ſmall funnel, or a tea-pot with a long neck, which reached beyond the injury in the palate.

He drew his breath with difficulty, though leſs ſo than from the nature of the caſe might have been expected.

The next day there was a conſiderable degree of fever, for which ſuitable remedies were directed. Care was taken at the ſame time to obviate coſtiveneſs; and the opiate was occaſionally repeated with a view to leſſen the effects of irritation.

On the ſixth day, as the bandages and dreſſings had become diſagreeable from the diſcharge, they were carefully removed. The pus was found to be moderate in quantity, and of a good conſiſtence; the wounds looked well; and the parts, notwithſtanding they had been much bruiſed, had already begun to unite. The ſame kind of dreſſing was applied, and in the ſame manner as before.

On

On the ninth day from the accident the dref-fing was again repeated. I had then the plea-fure to find the union in feveral places fuffi-ciently complete to infure a retention of the adjacent parts with a bandage lefs ftrict than the one that had at firft been applied. The wounds continued to look well, and to dif-charge a good pus; the patient was free from much pain, except a little upon the application of the dreffings: he refted well, and began to take confiderable nourifhment, the febrile fymp-toms gradually fubfiding.

From this time we began to drefs the wounds every day, and continued fo to do till the month of December following, when the whole (except a wound upon the external orbit of the injured eye) was firmly cicatrifed. A number of exfoliations and fome fmall fplinters of bone came away from this wound on the orbit, and occafioned it to be kept open a confiderable time; as was alfo a large orifice in the palate, from whence a confiderable exfoliation, that feemed to be a portion of the feptum nafi, came away.

From the time of the accident the patient loft the fight of the eye on the injured fide; and the decay of the other was gradual, without out any inflammation or pain. In other respects he is perfectly well.

